# Electro-Amputation of Lower Limbs Due to a High-Voltage Shock: Report of an Unusual Case

**DOI:** 10.7759/cureus.53808

**Published:** 2024-02-07

**Authors:** Suraj Sundaragiri, Senthil Kumaran M, Venkatesh Janarthanan, Chaitanya Mittal, Gerard Pradeep Devnath S

**Affiliations:** 1 Forensic Medicine, Gandhi Medical College, Secunderabad, IND; 2 Forensic Medicine & Toxicology, All India Institute of Medical Sciences, Madurai, Madurai, IND; 3 Forensic Medicine, All India Institute of Medical Sciences, Kolkata, Kolkata, IND; 4 Forensic Medicine & Toxicology, Dr BC Roy Multi Speciality Medical Research Centre, Indian Institute of Technology Kharagpur, Kharagpur, IND

**Keywords:** work-related death, bilateral lower limb amputation, electrical injury, electrical burns, high-voltage electrocution

## Abstract

Electrical injuries due to high voltage are not frequent but can cause a high mortality rate. The body of a 45-year-old security guard was found at an apartment with an alleged history of being electrocuted while working when he came into contact with a high-voltage transformer. The police brought the body for a post-mortem examination. The autopsy revealed superficial to deep-degree burns with bilateral lower limb amputation. The cause of death was shock due to 80%-85% of the total body surface area being burned due to high-voltage electrocution. This article suggests a new mechanism, i.e., electrocution-induced amputation, called electro-amputation, which is unusual and not reported in the literature to the best of our knowledge. The authors also recommend a forensic surgeon should consider detailed circumstantial findings, a scene of crime visit, and a meticulous post-mortem examination before concluding the exact cause of death as high-voltage electrocution.

## Introduction

Electrocution refers to an electric current that passes through the body and causes devastating injuries or death to individuals in an unguarded form. Because of modernization and commercial utilization, the requirement for electricity usage is increasing daily, which in turn is leading to an increase in deaths due to electrocution in India [1]. According to the National Electrical Code, electric injuries and deaths can occur either due to low voltage (< 600 V) or high voltage (> 600 V), and most of these incidents are accidental and preventable [2]. Most electrocuted-related deaths occur during the usage of electrical household appliances or construction equipment or the careless handling of live electric wires by an electrician in India, i.e., alternative currents of 220-240 volts with 50 amperes for domestic purposes [3].

High-voltage electrical injuries are comparatively infrequent and have a high mortality rate; however, they can noticeably contribute to occupational fatalities during work tasks [4]. The high morbidity seen in survivors depends upon the voltage strength that determines the destructive effects like electric burns to limbs leading to septicemia, increased toxin, and myoglobin released into the bloodstream causing life-threatening complications or even death [5]. To save the person, surgical amputation is the preferred treatment for this condition and is found in much literature [6]. In this article, the authors report a unique case of an adult male who sustained bilateral lower limb amputation following accidental contact with an electric transformer. This article suggests a new mechanism, i.e., electrocution-induced amputation, called electro-amputation, which is not available in the literature.

## Case presentation

**Figure 1:**
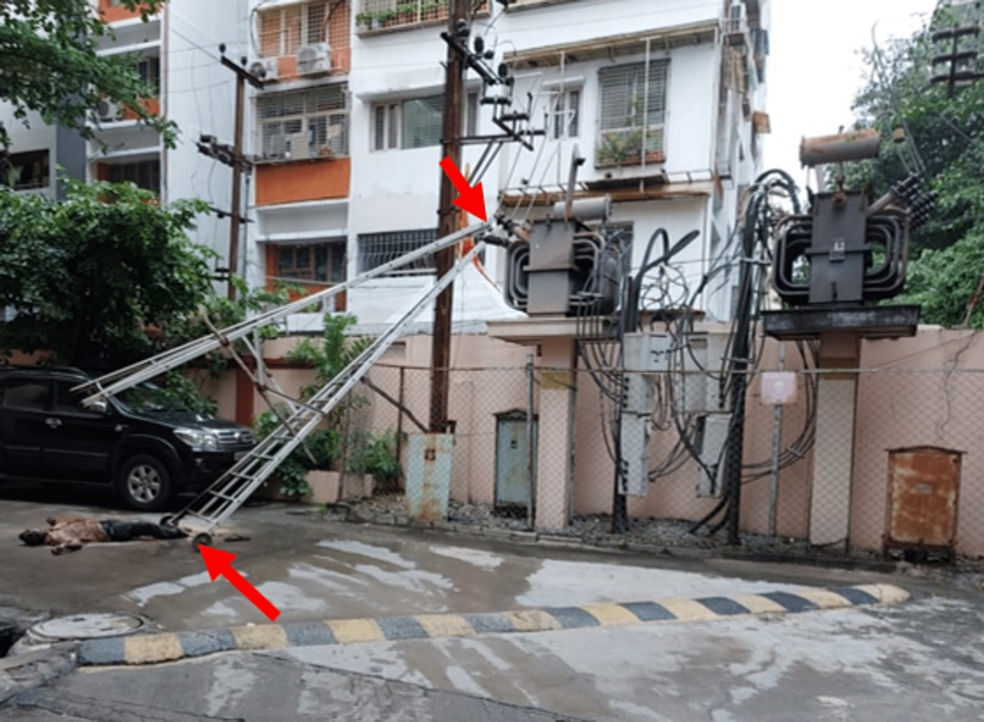
The crime scene shows the body on the ground, with both feet making contact with the ladder

The body of a 45-year-old male, a security guard at the apartment, was brought for a post-mortem examination to the mortuary of a tertiary care hospital. According to the investigating police officer, the victim was doing maintenance work at the apartment using an aluminum foldable (trolley step ladder) fixed with wheels at the bottom for easy moving. He also noted a speed breaker near the transformer inside that apartment. The victim was wandering with the ladder around the apartment at the time of the incident. While moving the ladder, he reached a spot where the speed breaker was, so he wanted to control the speed of the moving ladder. In the process of stopping the ladder with the help of wheels, the ladder wheel hit the speed breaker, lost control, and accidentally fell on the high-voltage transformer. Due to this, the victim suffered a high-tension electrical shock and died on the scene (Figure 1).

**Figure 2:**
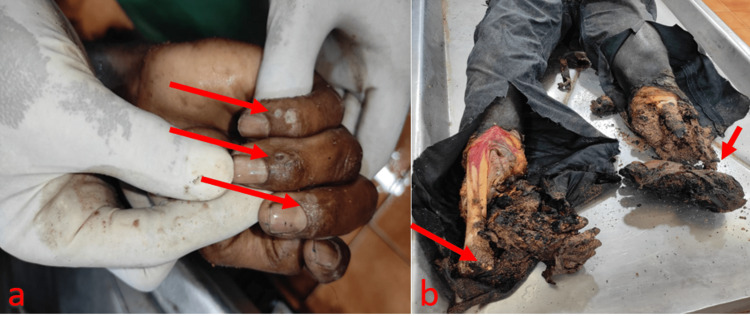
a: Joule burns present over the dorsal side of the right hand’s distal phalanges involving the middle, ring, and little fingers; b: Both lower limbs were completely charred with visible dry bones and amputation at ankle level (right side) and knee level (left side)

The autopsy revealed that the deceased sustained superficial to deep burns of about 80%-85% over the backside of the body, right side of the arm, forearm, chest, lower limbs, pelvis, and external genitalia. An electrical injury mark, consistent with a joule burn, was seen on the dorsal side of the right hand’s distal phalanges involving the middle, ring, and little fingers (Figure 2a). Deep burns involving the right and left legs with amputation were noted at the level of the ankle joint and below the knee, respectively. The amputation of both lower limbs at the level of the ankle joint showed charred, burnt edges, with exposure of muscles and bones (Figure 2b). The cause of death is due to burns involving 80-89% of the body surface due to high-voltage electrocution.

## Discussion

The incidence of high-voltage electrical injury is considerably lower than other electrical injuries; however, the fatality rate is very high with direct contact, indirect arcing, or flashover effects. Similar to our case, most of the fatalities reported were among manual workers [4]. Sometimes, diagnosing high-voltage electrocution without history and circumstantial evidence becomes difficult. This is because features that are considered pathognomonic of electrocution, like electric marks and joule burns, are often seen only with low or medium voltage current, and the involvement of both entry and exit marks is seen together only in 20% of cases [7].

In our case, the person was in contact with the ladder, as he was holding it with his hands, which fell on the transformer due to a current passage that caused the joule’s burn within a point of contact, i.e., the hand point of entry. Following this, arcing occurs in high-voltage electricity by creating plasma conduction between the source and ground, leading to the blast effect of high-voltage electric arcing. That causes the person to be thrown away from the source and lose his balance, leading to the entrapment of his leg between the ladder and the ground. The current passage from the transformer through the ladder to the legs leads to electrothermal heating, generating temperatures of up to 4000°C and more, resulting in extensive tissue damage along the current flow path. High-voltage electrical injuries can lead to extensive necrosis, subsequent tissue loss, severe damage to underlying structures (muscles, nerves, blood vessels, and bones), and burns into ashes, resulting in electric current-induced amputations, i.e., electro-amputation with visible dry bones [8-10].

Multiple sparks burn in the victim cause a large area of tissue damage and give rise to a ‘crocodile skin’ appearance. Here, the patient sustained high-tension electrical burns with associated secondary flame burns due to the ignition of clothes, which is documented to be common [11].

As observed in various instances, high-voltage casualties are due to the unsafe proximity of residential buildings to high-voltage electrical lines and transformers, breaching minimum clearance standards. In urban areas, numerous facilities are situated improperly, often putting the lives of occupants in danger at any given time. Efforts by regulatory authorities in multiple disciplines should enforce minimum safe clearance of residential buildings, and public enlightenment will go a long way in preventing high-voltage electric injuries. 

## Conclusions

Reporting of incidences due to high-voltage electrocution with lower limb amputation is somewhat rarer than other electrical injuries. This case emphasizes the need to wear personal protective equipment and maintain proper interpersonal communication during work hours as per the Indian Electricity Rules 1956. This article recommends that forensic surgeons consider detailed circumstantial findings, a crime scene visit, and a meticulous post-mortem examination before concluding the exact cause of death due to high-tension electrocution.
